# Expression of FOXI1 and POU2F3 varies among different salivary gland neoplasms and is higher in Warthin tumor

**DOI:** 10.1007/s12672-024-00892-7

**Published:** 2024-02-15

**Authors:** Masahito Hoki, Yosuke Yamada, Emi Hiratomo, Masahiro Hirata, Yasuhide Takeuchi, Masayoshi Yoshimatsu, Masahiro Kikuchi, Yo Kishimoto, Alexander Marx, Hironori Haga

**Affiliations:** 1 Department of Diagnostic Pathology, Kyoto University Hospital, 54 Shogoin Kawahara-cho, Sakyo-ku, Kyoto, 606-8507 Japan; 2 Department of Otolaryngology, Head and Neck Surgery, Graduate School of Medical and Dental Sciences, Kagoshima University, Kagoshima, Japan; 3 Department of Otolaryngology-Head & Neck Surgery, Kobe City Medical Center General Hospital, Kobe, Japan; 4 Department of Otolaryngology, Head and Neck Surgery, Graduate School of Medicine, Kyoto University, Kyoto, Japan; 5 Institute of Pathology, University Medical Center Göttingen, University of Göttingen, Göttingen, Germany

**Keywords:** POU2F3, FOXI1, Tuft cells, Ionocytes, Salivary gland neoplasms, Warthin tumor

## Abstract

**Purpose:**

Salivary gland tumors are histologically diverse. Ionocytes and tuft cells, rare epithelial cells found in normal salivary glands, might be associated with salivary tumors. Here, we explored the expression of FOXI1 and POU2F3, master regulators of ionocytes and tuft cells, respectively, for common salivary neoplasms using immunohistochemistry.

**Methods:**

We analyzed normal salivary tissues and nine salivary gland tumors; Warthin tumors (WT), pleomorphic adenomas (PA), basal cell adenomas, and oncocytomas were benign, whereas mucoepidermoid, adenoid cystic, acinic cell, salivary duct carcinomas, and polymorphous adenocarcinomas were malignant.

**Results:**

Normal salivary glands contained a few FOXI1- and POU2F3-positive cells in the ducts instead of the acini, consistent with ionocytes and tuft cells, respectively. Among the benign tumors, only WTs and PAs consistently expressed FOXI1 (10/10 and 9/10, respectively). The median H-score of WTs was significantly higher than that of PAs (17.5 vs. 4, *P* = 0.01). While WTs and PAs harbored POU2F3-positive cells (10/10 and 9/10, respectively), the median H-score was higher in WTs than in PAs (10.5 vs 4, respectively). Furthermore, WTs exhibited a unique staining pattern of FOXI1- and POU2F3-positive cells, which were present in luminal and abluminal locations, respectively. Whereas none of the malignant tumors expressed FOXI1, only adenoid cystic carcinoma consistently expressed POU2F3 (5/5), with a median H-score of 4.

**Conclusion:**

The expression patterns of the characteristic transcription factors found in ionocytes and tuft cells vary among salivary gland tumor types and are higher in WT, which might be relevant for understanding and diagnosing salivary gland neoplasms.

## Introduction

Salivary gland tumors, which occur in both major and minor salivary glands, display remarkable histological variety and are divided into many subtypes. Several subtype can be associated with tumor-specific rearrangements, such as *CRTC1::MAML2* in mucoepidermoid carcinoma, *MYB::NFIB* or *MYBL1::NFIB* in adenoid cystic carcinoma, and *ETV6::NTRK3* in secretary carcinoma. These features facilitate pathological diagnoses and clinical management [1]. However, even genetically defined tumors can be morphologically diverse within a single subtype, and not all subtypes have specific genetic abnormalities. Thus, the (immuno-)phenotypes associated with subtypes or cytomorphology can provide a better understanding of salivary gland tumors. Further, tumors typical of salivary glands, or salivary gland-type tumors, also occur in other organs [2], suggesting the importance of studying these neoplasms. This can advance our comprehensive knowledge of human neoplasia.

Single-cell RNA sequencing studies and subsequent functional assays have revealed the presence and significance of rare and previously under-recognized cell types, such as ionocytes and tuft cells. Human ionocytes were first reported in the lung [3, 4], where they regulate airway surface physiology by expressing characteristic functional molecules, such as cystic fibrosis transmembrane conductance regulator (CFTR) [5], governed by the master regulator Forkhead Box I1 (FOXI1) [3, 4]. Tuft cells, which are epithelial cells characterized by unique microvilli (tufts) on their apical side, are present in many organs [6–9]. These cells are involved in type 2 immunity and initiate antiparasitic immune responses in the intestines [10–12].

Ionocytes and tuft cells are physiologically present in salivary glands. Among several studies on salivary gland ionocytes [13–15], Mauduit et al. revealed that these cells not only maintain the specific ion composition in the saliva but also function as niche cells that support other epithelial cells by providing growth factors, especially fibroblast growth factor (FGF) 10 [15]. This “niche” function of ionocytes expands its biological significance. Further, Tavares dos Santos et al. demonstrated the presence of tuft cells in submandibular glands across species [6]. To our knowledge, the comprehensive function of salivary gland tuft cells has not been addressed yet.

These two epithelial cell types, particularly tuft cells, have recently attracted attention in cancer research, especially after the discovery of a tuft cell-like variant of small cell lung cancer (SCLC). This variant exhibits a signature tuft cell-like gene expression pattern, including POU class 2 homeobox 3 (POU2F3), a tuft cell master regulator [7, 16]. Subsequently, carcinomas with tuft cell-like expression profiles were discovered in extrapulmonary organs [17–21]. Interestingly, these tuft cell-like carcinomas shared ionocyte-like phenotypes, including the expression of FOXI1 [19, 22]. They often exhibit high-grade histology and significantly express well-known oncogenes, including receptor tyrosine kinase (KIT) and B-cell lymphoma 2 (BCL2). Moreover, tuft cell-like carcinomas have been shown to exhibit unique sensitivity toward drugs, such as poly (ADP-ribose) polymerases (PARP) inhibitors [19, 22, 23].

We speculated that tumors with ionocyte- or tuft cell-like phenotypes might be present in previously unexamined organs and tumor types. In this study, we tested this hypothesis for salivary gland tumors using immunohistochemistry (IHC) for FOXI1 and POU2F3 because, as mentioned, both ionocytes and tuft cells are found in salivary glands. These glands also display histologically diverse tumors, and we hypothesized that some might exhibit the phenotypes of rare epithelial cell subsets.

## Materials and methods

### Case selection

We selected 53 cases of nine types of common salivary gland tumors from the archives of Kyoto University Hospital between 1992 and 2021. Among these, Warthin tumors (WT), pleomorphic adenomas (PA), basal cell adenomas, and oncocytomas were benign, while mucoepidermoid, adenoid cystic, acinic cell, and salivary duct carcinomas, and polymorphous adenocarcinomas were malignant. We first retrieved the five most recently archived cases of each tumor type, except oncocytoma, as our archives had only three cases. All cases were reviewed by two pathologists (M.H. and Y.Y.). The already available histological slides (stained using hematoxylin and eosin and IHC) were used and the original pathological diagnoses were reconfirmed. Because only WTs and PAs consistently or frequently expressed both FOXI1 and POU2F3, we expanded the numbers of samples of these two types from five to ten in the same manner. Clinical findings of patients were obtained from medical records. We also evaluated the non-neoplastic salivary glands, including parotid (N = 27), submandibular (N = 5), sublingual (N = 1), and minor (N = 11) around the tumors (when available) or within the biopsy specimens.

Aside from these cases, we enrolled all PAs (N = 2) and mucoepidermoid carcinomas (N = 1) with prominent oncocytic changes from the above archive. However, as these three cases were not statistically analyzed, they were not included in Tables 1, 2.

**Table 1:** Clinical findings of nine types of salivary gland tumors in patients

Tumor type	No	Age	Female	Smoker^a^	Pack	Year	Pack-Year	Tumor site^b^				
		(median)	(no.)	(no.)	(median)	(median)	(median)	P (no.)	Sm	Sl	M	O
Benign tumors												
Warthin tumor	10	67.5	2	9	0.68	42.5	25.8	10	0	0	0	0
Pleomorphic adenoma	10	49.5	9	4	0	0	0	6	1	0	2	1
Basal cell adenoma	5	73	2	3	0.25	6	1.5	5	0	0	0	0
Oncocytoma	3	58	2	0	0	0	0	3	0	0	0	0
Malignant tumors												
Mucoepidermoid carcinoma	5	35	3	1	0	0	0	1	0	0	4	0
Adenoid cystic carcinoma	5	59	4	2	0	0	0	0	3	1	1	0
Acinic cell carcinoma	5	69	2	2	0	0	0	4	0	0	1	0
Polymorphous adenocarcinoma	5	66	3	2	0	0	0	0	0	0	5	0
Salivary duct carcinoma	5	69	1	4	0.5	6	6	4	1	0	0	0

^a^Current or past smokers

^b^*P* parotid gland, *Sm* submandibular gland, *Sl* sublingual gland, *M* minor salivary gland, *O* other sites

**Table 2:** Results of FOXI1- and POU2F3-immunohistochemistry for nine salivary gland tumor types

Tumor type	N	FOXI1-positive cells					POU2F3-positive cells				
		Present	Absent	*P*-value^a^	H-score^b^	*P*-value^c^	Present	Absent	*P*-value	H-score	*P*-value
Benign tumors	28	20	8				22	6			
Warthin tumor	10	10	0	–	17.5	–	10	0	–	10.5	–
Pleomorphic adenoma	10	9	1	0.30	4	0.01	9	1	0.30	4	0.42
Basal cell adenoma	5	1	4	0.004	0	0.004	2	3	0.02	0	0.04
Oncocytoma	3	0	3	0.004	0	0.01	1	2	0.04	0	0.06
Malignant tumors	25	0	25				9	16			
Mucoepidermoid carcinoma	5	0	5	< 0.001	0	0.002	1	4	0.004	0	0.004
Adenoid cystic carcinoma	5	0	5	< 0.001	0	0.002	5	0	1.00	4	0.38
Acinic cell carcinoma	5	0	5	< 0.001	0	0.002	0	5	< 0.001	0	0.002
Polymorphous adenocarcinoma	5	0	5	< 0.001	0	0.002	0	5	< 0.001	0	0.002
Salivary duct carcinoma	5	0	5	< 0.001	0	0.002	3	2	0.10	4	0.29

^a^Difference of frequency vs. Warthin tumor (Chi-square test or Fisher’s exact test)

^b^Median H-score (the percentage of immunoreactive cells (0%–100%) x the intensity of labeling [1, weak; 2, moderate; 3, strong])

^c^Difference of H-score vs. Warthin tumor (Wilcoxon test)

### Immunohistochemistry

IHC was performed on formalin-fixed, paraffin-embedded specimens using an automated immunostainer (Benchmark Ultra, Ventana Medical Systems, Oro Valley, AZ, USA). One representative slide per case was examined. The primary antibodies were against FOXI1 (rabbit polyclonal, Atlas Antibodies, Bromma, Sweden) and POU2F3 (E5N2D, Cell Signaling Technology, Danvers, MA, USA). Renal tubules and skin keratinocytes were used as positive controls for FOXI1 and POU2F3, respectively [24, 25]. Only nuclear staining was considered positive because both proteins are nuclear transcription factors [24, 25]. The results were evaluated using H-scores, a common method for immunohistochemical semi-quantification [26, 27]. The value is determined by multiplying the estimated percentage of immunoreactive cells (0–100%) by the labeling intensity (1, weak; 2, moderate; 3, strong), thus ranging from 0 to 300; it is reported to be correlated with scores obtained by biological assays [26, 27].

For particular cases of WTs, we also performed IHC for BCL2 (SP66, Roche diagnostics, Basel, Switzerland), KIT (polyclonal, Agilent Technologies, Santa Clara, CA, USA), p63 (7JUL, Leica Biosystems, Wetzlar, Germany), p40 (BC28, Roche diagnostics), alpha smooth muscle actin (aSMA) (1A4, Sigma-Aldrich, St. Louis, MO, USA), and calponin (CALP, Agilent Technologies), to address whether POU2F3-positive cells within the WTs co-expressed BCL2 and KIT as typical tuft cell-like carcinomas [19] and/or p63, p40, aSMA, and calponin, which are abluminal markers.

### Statistical analysis

Differences in the categorical variables were evaluated using the Chi-square or Fisher’s exact test (the latter was used when cells with the expected values of < 5 exceeded 20%), while those in the continuous variables were compared by the Wilcoxon test. Differences at *P* < 0.05 were considered significant. All statistical analyses were performed using the JMP17 software (Statistical Analysis System, Cary, NC, USA).

## Results

### Clinical findings

The number of male and female patients was 25 and 28, respectively. The patients’ ages ranged from 21 to 84 years (median age = 59 years). In addition to sex and age, Table 1 shows the smoking status and tumor site according to each histological subtype. As expected, nine out of ten patients with Warthin tumor had a smoking history, with a median of 25.8 pack-years.

### Presence of FOXI1- and POU2F3-positive cells in normal salivary glands

**Fig. 1:**
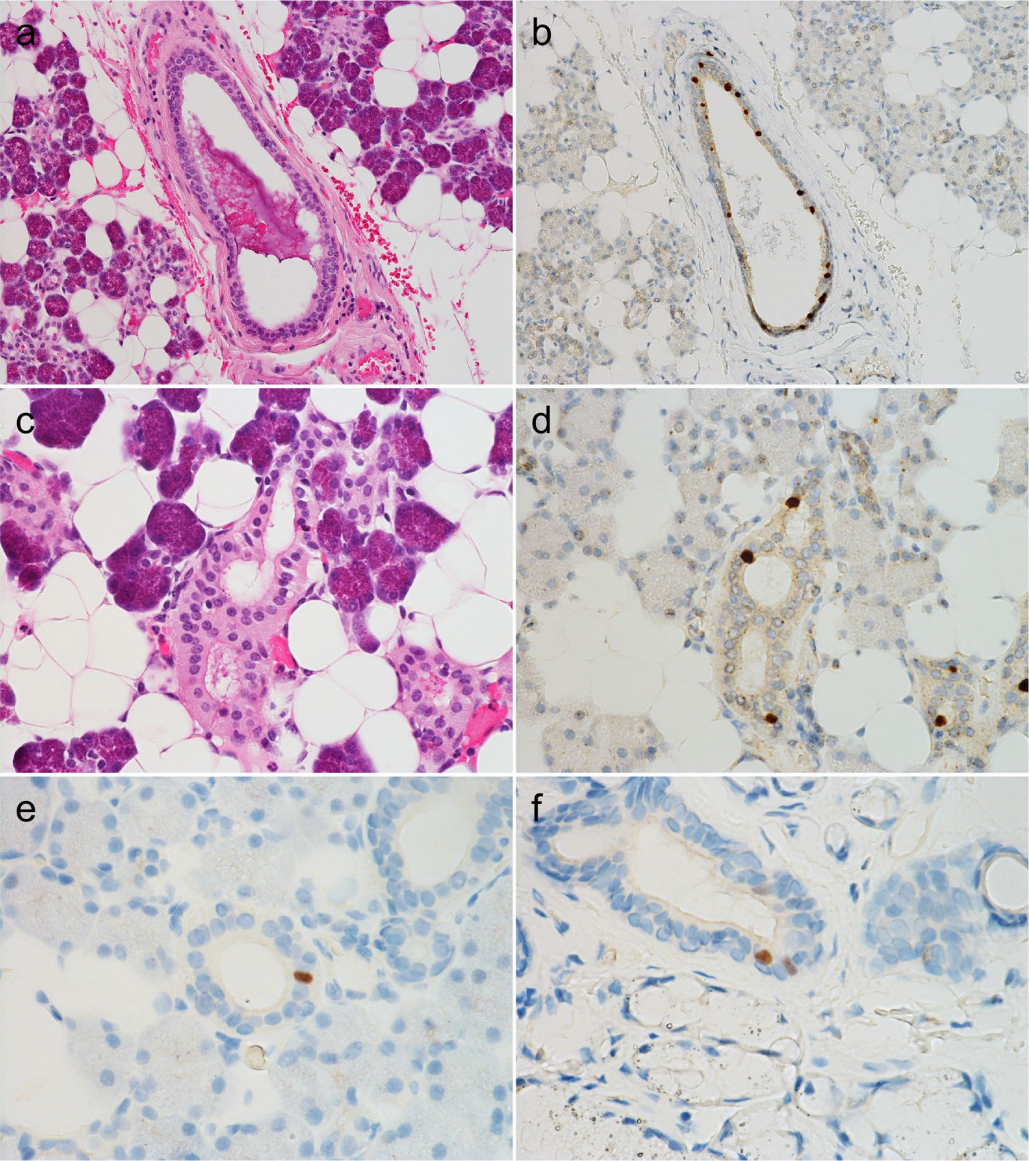
FOXI1- and POU2F3-positive cells in non-neoplastic parotid and submandibular glands. **a**–**e** Parotid and (**f**) minor salivary glands. FOXI1-positive cells were observed in interlobular (**b**), striated (**c**), and intercalated (**d**) ducts. While POU2F3-positive cells were primarily seen in the striated duct (**e**) and the luminal layer (**e**, **f**), some were found in the abluminal layer (arrow) (**f**) (**a**, **c**: hematoxylin and eosin staining; **b**, **d**-**f**: immunohistochemistry [**b**, **d**: FOXI1, **e**, **f**: POU2F3])

Initially, we evaluated the expression of FOXI1 and POU2F3 in normal salivary glands (parotid, submandibular, sublingual, and minor) around tumors or within biopsy specimens. We found that all these glands contained a few FOXI1- and POU2F3-positive cells. The FOXI1-positive cells constituted < 5% of all epithelial cells and were located mainly in interlobular and striated ducts, rarely in intercalated ducts, but never in acini (Fig. 1a–d). FOXI1-positive cells were always located on the luminal side, not the abluminal side, when two layers were discernible (Fig. 1a–d). POU2F3-positive cells were very rare (< < 1% of the epithelial cells) and were primarily observed in striated ducts and never in acini. They were observed mainly on the luminal side but rarely on the abluminal side (Fig. 1e, f).

### FOXI1 and POU2F3 expression in benign salivary tumors: the uniqueness of Warthin tumor

Next, we examined FOXI1 and POU2F3 expression in common benign salivary gland tumors: WTs, PAs, basal cell adenomas, and oncocytomas. FOXI1-IHC showed that while all the WTs (10/10) and most of the PAs (9/10) contained FOXI1-positive cells, these cells were never observed in basal cell adenomas and oncocytomas, except for one basal cell adenoma that had a few FOXI1-positive cells (Table 2).

**Fig. 2:**
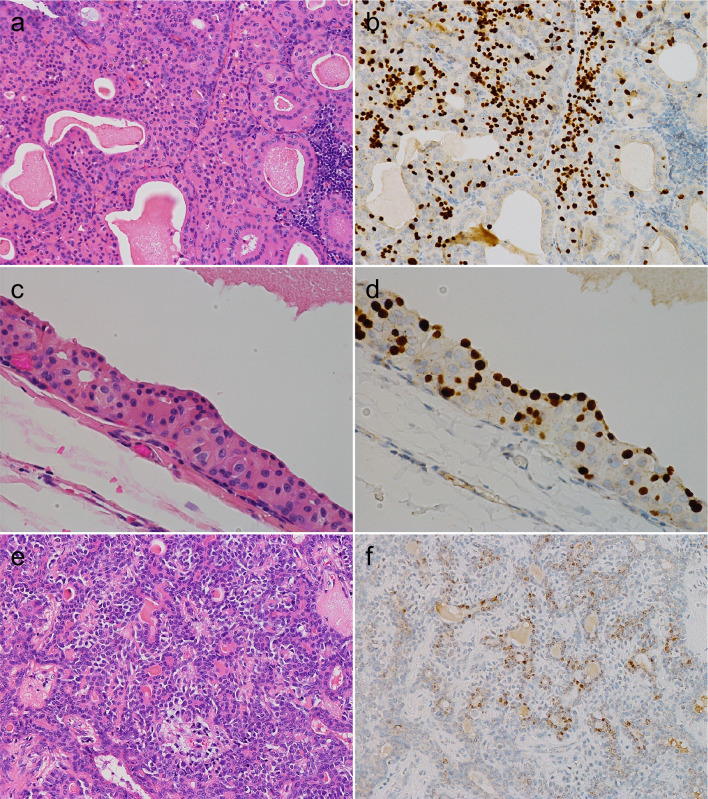
FOXI1 expression in Warthin tumor and pleomorphic adenoma. **a**–**d** Warthin tumor, (**e**, **f**) pleomorphic adenoma. Warthin tumor contained many strongly FOXI1-positive cells (**a**, **b**), which had slightly smaller cytoplasms than surrounding tumor cells and were located between/among the inner lining cells when the tumor formed cystic structures (**c**, **d**). Focal and weak expression of FOXI1 was observed in the duct-forming areas of pleomorphic adenoma (**a**, **c**, **e**: hematoxylin and eosin staining; **b**, **d**, **f**: immunohistochemistry)

**Fig. 3:**
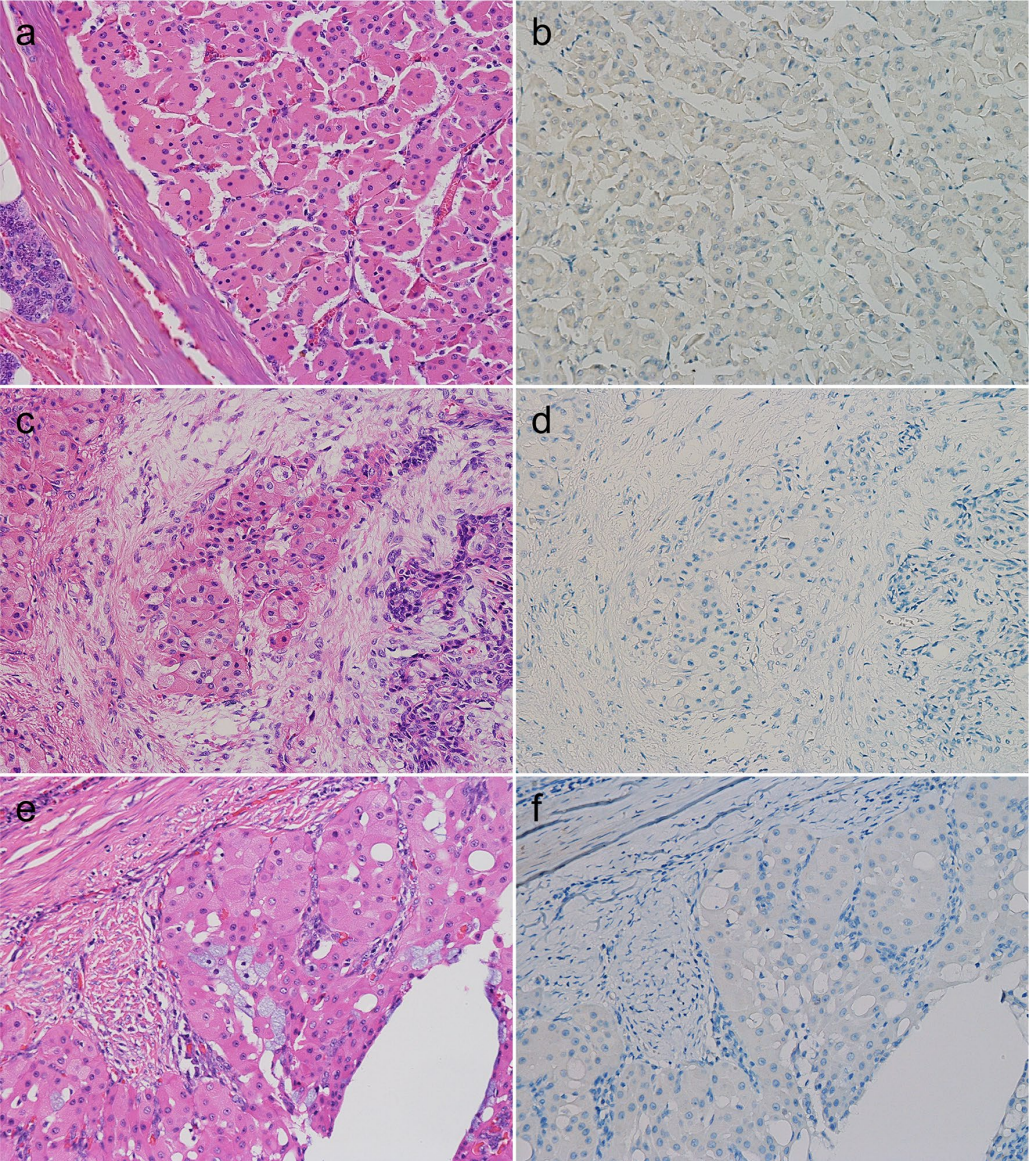
FOXI1 immunohistochemistry in oncocytoma, oncocytic pleomorphic adenoma, and oncocytic mucoepidermoid carcinoma. All cases of (**a**, **b**) oncocytoma, (**c**, **d**) oncocytic pleomorphic adenoma, and (**e**, **f**) oncocytic mucoepidermoid carcinoma were negative for FOXI1 (**a**, **c**, **e**: hematoxylin and eosin staining; **b**, **d**, **f**: immunohistochemistry)

WTs and PAs had distinct staining patterns. In WTs, FOXI1-positive cells were relatively broadly distributed, and the staining intensity was generally strong (Fig. 2a, b). The FOXI1-positive cells in the WTs appeared to have slightly less cytoplasm than the surrounding tumor cells and were located on the luminal side when the tumor formed cystic or glandular structures (Fig. 2c, d). In PAs, FOXI1-positive cells generally occurred focally among luminal cells in duct-forming areas, and the staining intensity was often weak to moderate (Fig. 2e, f). As WTs are characterized by their oncocytic morphology, we performed IHC for three salivary gland tumors with oncocytic changes (pleomorphic adenoma [N = 2] and mucoepidermoid carcinoma [N = 1]). Similar to oncocytoma, they were negative for FOXI1 (Fig. 3a–f).

**Fig. 4:**
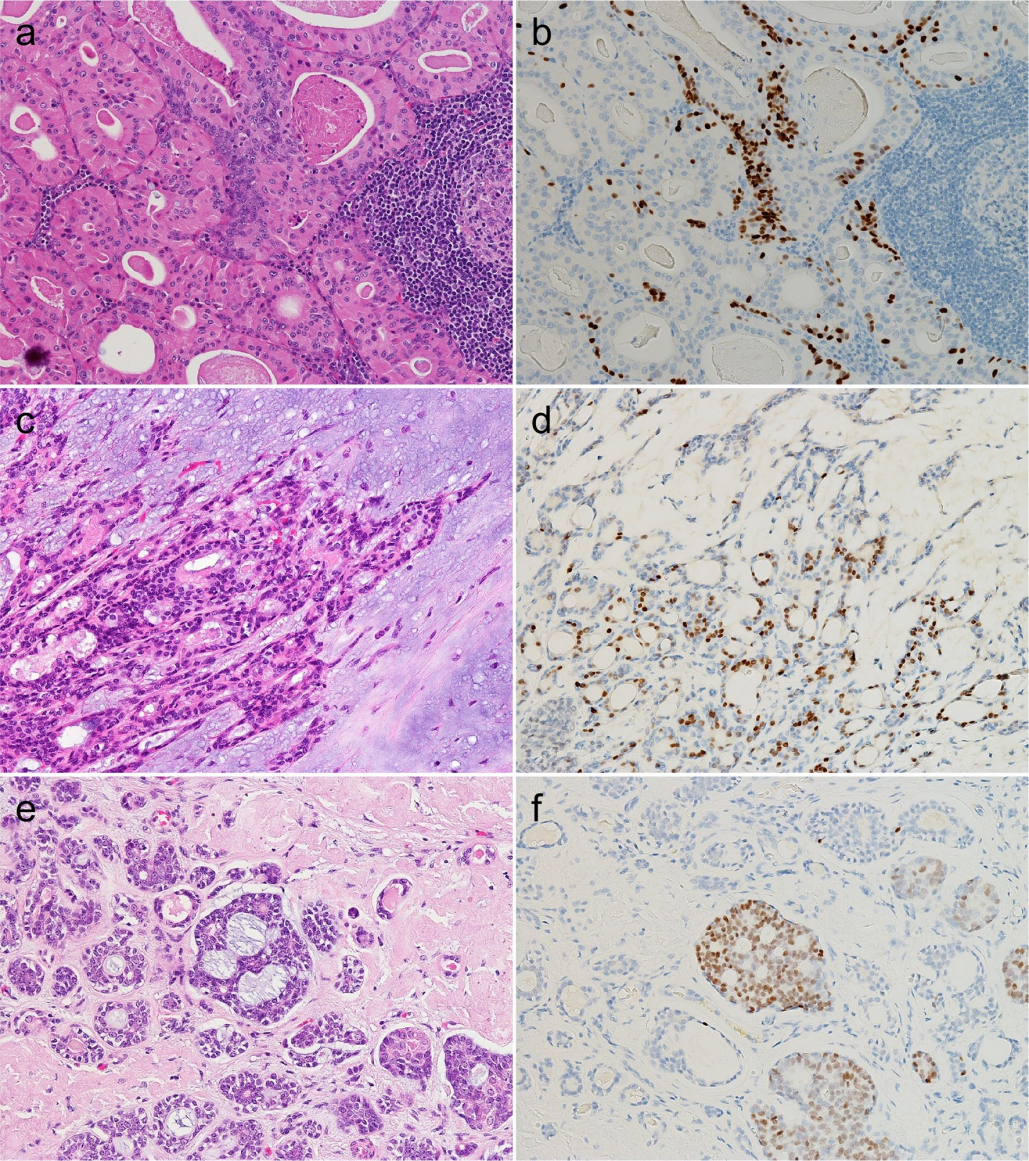
POU2F3 expression in Warthin tumor, pleomorphic adenoma, and adenoid cystic carcinoma. **a**, **b** Warthin tumors, (**c**, **d**) pleomorphic adenoma, (**e**, **f**) adenoid cystic carcinoma. Warthin tumors contained many POU2F3-positive cells, which tended to be between/among the cells of the outer lining (when the two layers are visible as in a, b; see also Fig. 4). POU2F3-positive cells in pleomorphic adenomas were observed consistently in duct-forming epithelial components but not mesenchymal components. POU2F3-positive cells in adenoid cystic carcinomas were observed as small aggregates in a few nests (**a**, **c**, **e**: hematoxylin and eosin staining; **b**, **d**, **f**: immunohistochemistry)

**Fig. 5:**
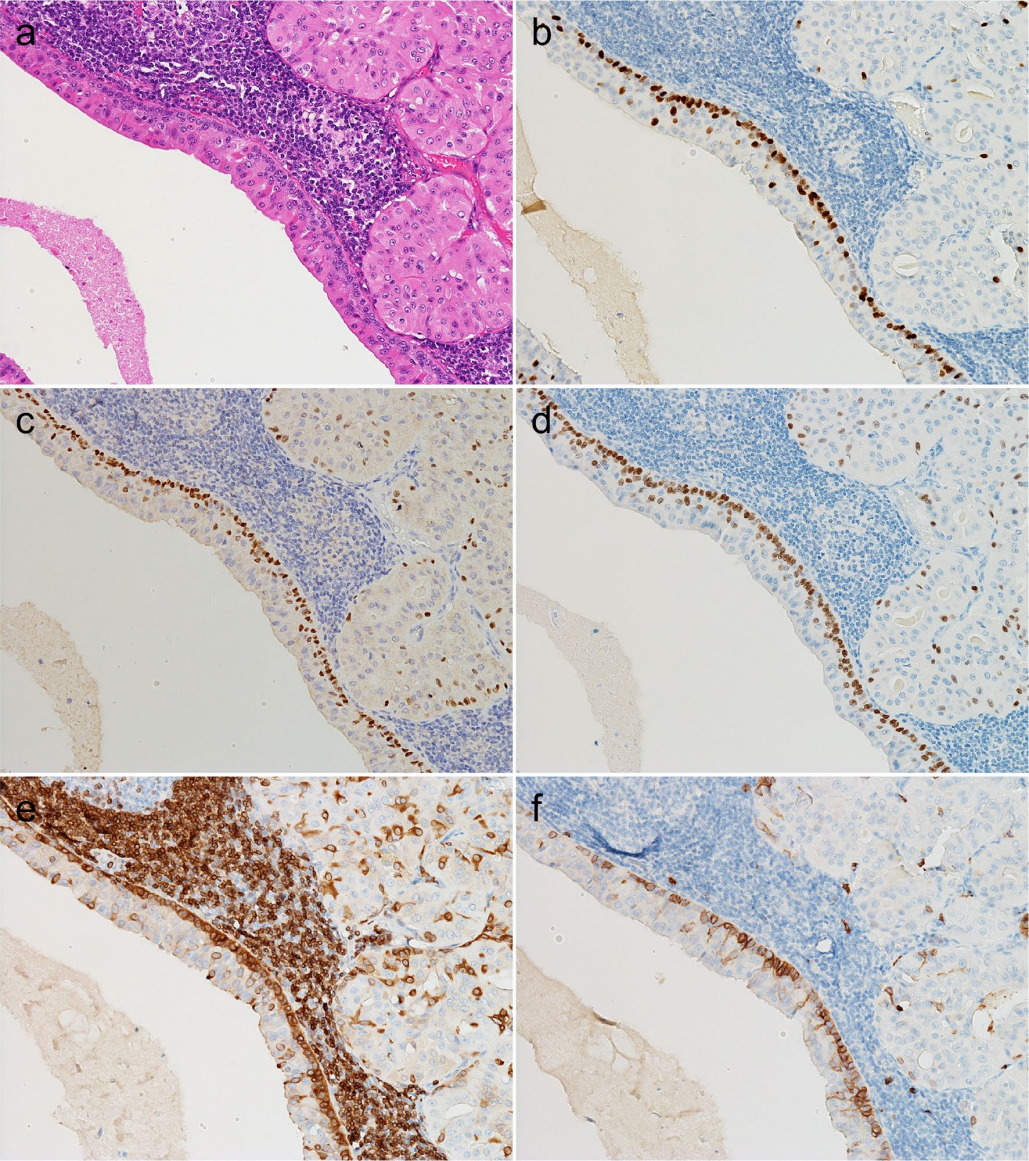
Coexpression of POU2F3, p63, and p40 in Warthin tumor. POU2F3-positive cells in Warthin tumors were found in abluminal layers and can coexpress p63 and p40, both common myoepithelial and basal markers, along with BCL2 and KIT. (**a**: hematoxylin and eosin staining; **b**–**f**: immunohistochemistry for POU2F3 [**b**], p63 [**c**], p40 [**d**], BCL2 [**e**], and KIT [**f**])

**Fig. 6:**
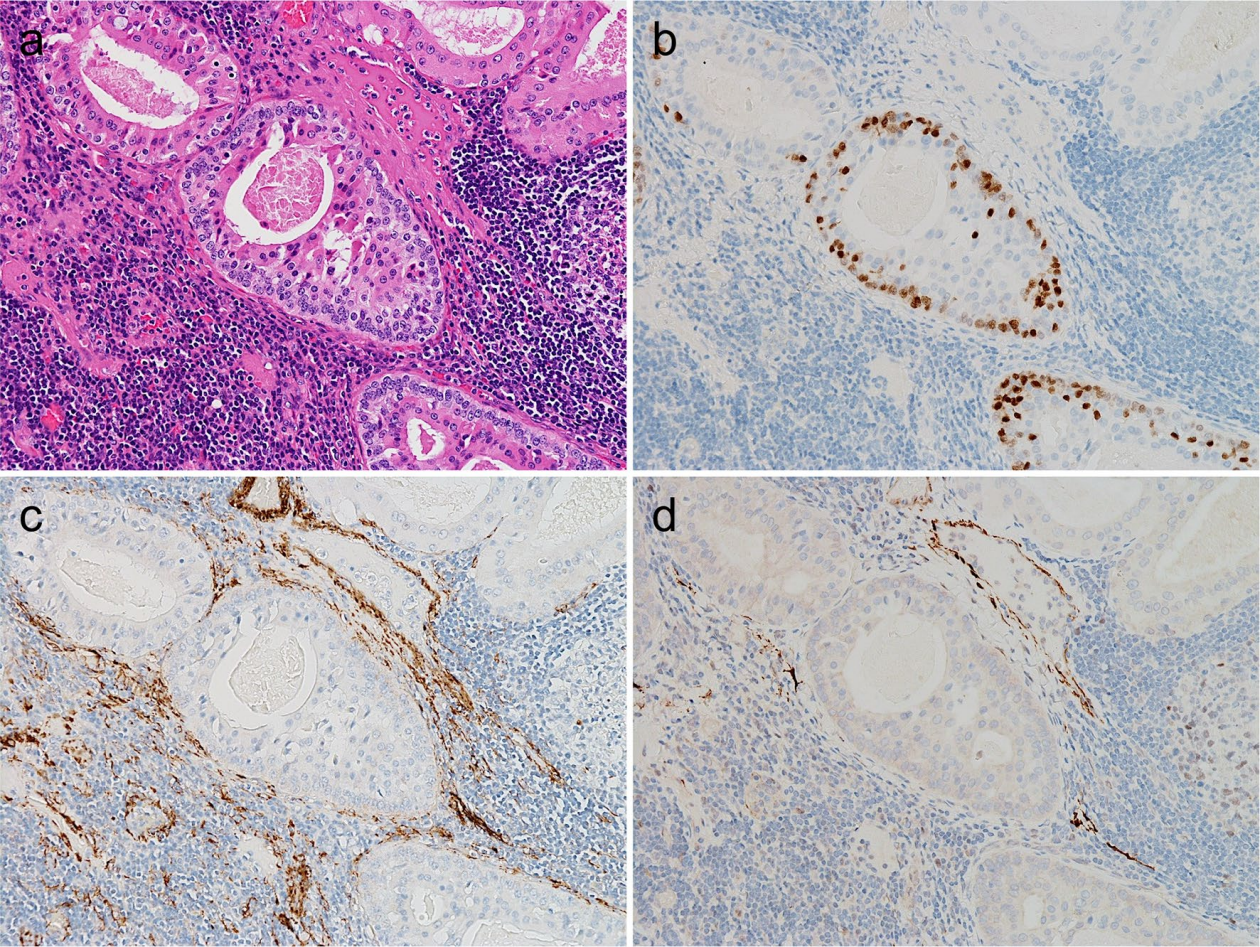
Lack of expression of aSMA and calponin in POU2F3-positive cells in Warthin tumor. POU2F3-positive cells in Warthin tumor did not express aSMA and calponin, common myoepithelial markers. (**a**: hematoxylin and eosin staining; **b**–**d**: immunohistochemistry for POU2F3 [**b**], aSMA [**c**], and calponin [**d**])

**Fig. 7:**
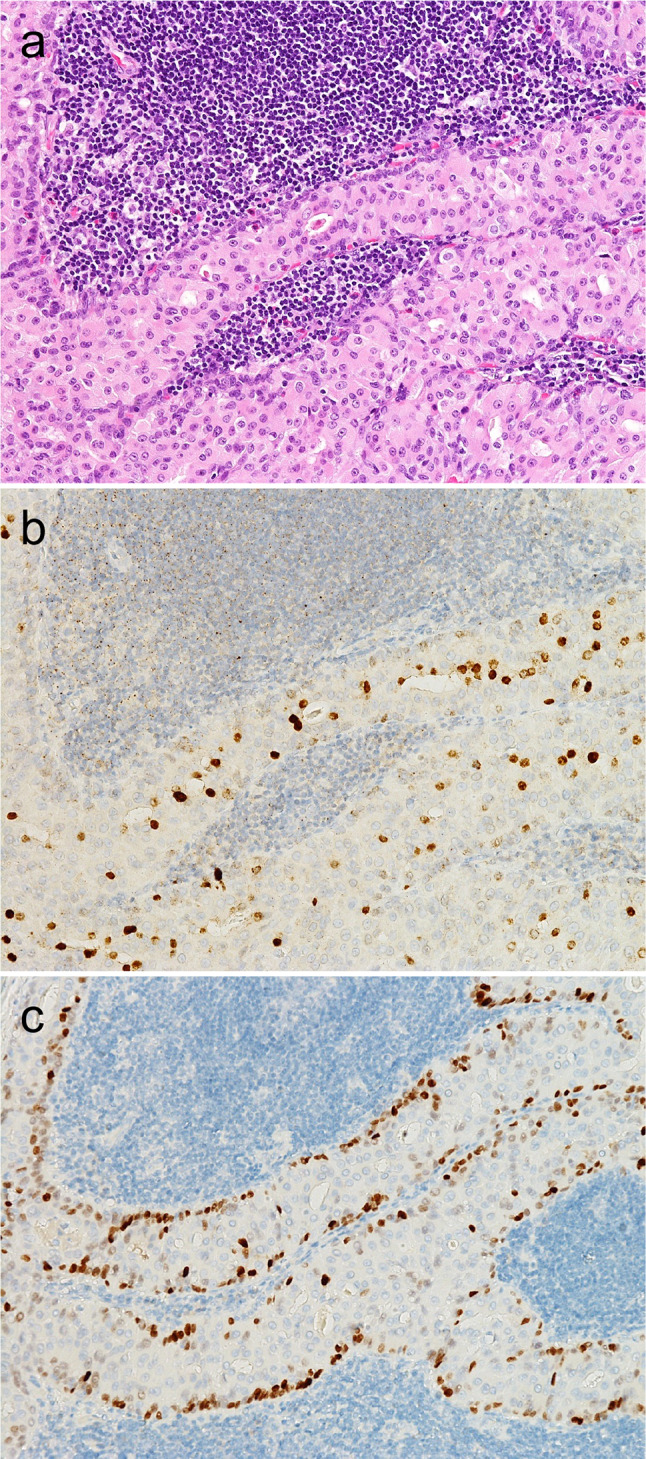
Differential FOXI and POU2F3 expression in Warthin tumor. Warthin tumor exhibited a unique biphasic pattern consisting of luminal FOXI1- and abluminal POU2F3-positive cells (**a**: hematoxylin and eosin staining; **b**, **c**: immunohistochemistry for FOXI1 [**b**], and POU2F3 [**c**])

Further, all or most WTs and PAs harbored POU2F3-positive cells (10/10 and 9/10, respectively). In addition, two basal cell adenomas (2/5) and one oncocytoma (1/3) also contained POU2F3-positive cells (Table 2). Although even WTs were not diffusely positive for POU2F3, each subtype exhibited different staining patterns. In WTs, POU2F3-positive cells tended to be on the abluminal side when two layers were recognizable and, at least partly, co-expressed p63 and p40, common abluminal markers, along with KIT and BCL2 (Figs. 4a, b and 5a–f), which are often expressed in carcinomas with tuft cell-like phenotype [17, 19, 22]. The POU2F3-positive cells were negative for aSMA and calponin, representative myoepithelial markers (Fig. 6a–d). Collectively, WTs exhibited a unique differential expression pattern of luminal FOXI1- and abluminal POU2F3-positive cells (Fig. 7). In PAs, POU2F3-positive cells were associated mostly with the luminal side of epithelial components but not with mesenchymal components (Fig. 4c, d).

### FOXI1 and POU2F3 expression in malignant salivary tumors

None of the cases of the five most common malignant salivary gland neoplasms, including mucoepidermoid, adenoid cystic, acinic cell, and salivary duct carcinomas, and polymorphous adenocarcinomas, contained FOXI1-positive cells (Table 2). The results of POU2F3-IHC varied. Only adenoid cystic carcinoma consistently expressed POU2F3 (5/5), however tumor cells were overall sparse and rarely formed homogeneously POU2F3-positive cell nests (Fig. 4e, f).

### Comparisons of FOXI1 and POU2F3 expression among different salivary gland tumors

Finally, we statistically compared the expression status of FOXI1 and POU2F3 among different salivary gland tumors. The expression of FOXI1, which were only seen in WTs and PAs, were significantly different between WTs and the other seven tumor types (*P* < 0.01) and between PAs and the other seven tumor types (*P* < 0.05; Table 2). As both WTs and PAs are benign, the frequency of FOXI1 expression differed significantly between benign and malignant tumors (20/28 and 0/25, respectively, *P* < 0.001; Table 2). In addition, the H-scores for the WTs were significantly higher than that of any other subtypes, ranging from 2 to 60 for WTs (median = 17.5), 0 to 15 for PAs (median = 4) (*P* = 0.01), and zero in the other seven subtypes (*P* < 0.01) (Table 2). The frequencies of POU2F3 expression also differed among subtypes and were significantly higher in benign than malignant tumors (22/28 vs. 9/25, *P* = 0.001; Table 2). The H-score of WT (median = 10.5) was the highest among the nine subtypes (Table 2).

## Discussion

We examined the expression status of transcription factors related to two rare epithelial cell types, ionocytes and tuft cells, in several commonly occurring salivary gland tumors. First, we observed minor populations of FOXI1- and POU2F3- positive cells in all normal major salivary glands, consistent with previous studies [6, 13, 14]. Based on this data, we assumed that the FOXI1- and POU2F3-positive cells in normal salivary glands correspond to ionocytes and tuft cells, respectively, and that IHC using FOXI1 and POU2F3 can be used to screen these rare cell types.

Among tumoral lesions, we found that FOXI1 and POU2F3 expression patterns were associated with different histotypes. The results in WTs were noteworthy as these tumors always harbored FOXI1 and POU2F3-positive cells with unique staining patterns: FOXI1 and POU2F3 were seen in the luminal and abluminal cells, respectively. Because the proportion of immunoreactive cells and the H-scores for FOXI1 and POU2F3 were generally low, even in WTs, we cannot state that the tumor cells in WTs diffusely express FOXI1 and POU2F3. Instead, we believe that WTs characteristically have the strongest capacity to produce FOXI1- and POU2F3-positive cells among common salivary gland tumors.

The reason behind this unique staining pattern of WTs is a fundamental question. WTs are benign and the second most common salivary gland tumor. The clinicopathological features are almost exclusive to the parotid gland and are associated with smoking [1]. These features were consistent with our WT patients, as all tumors occurred in the parotid gland, and nine out of ten patients had a long smoking history. Regarding pathogenesis, WTs probably arise from salivary duct inclusions in parotid lymph nodes through a reactive rather than a neoplastic process [1, 28–31].

Because smoking can be associated with phenotypic changes in the epithelial cells of WTs, such as damage to the mitochondrial genome [32, 33], we speculate that prolonged smoking might induce the peculiar differentiation propensity of WT cells. This hypothesis might be supported by studies on the lungs, which suggest that smoking can change cellular components, including rare cell types [34, 35]. In addition, a recent study demonstrated that lung injury can induce tuft cells with the basal phenotype (i.e., POU2F3^+^/p63^+^ cells) [36], which is similar to the POU2F3-positive cells in the abluminal, p63-positive layer in WTs. Our results that the number of physiological POU2F3-positive cells in normal salivary glands was minimal and mostly present among the luminal cells may suggest that POU2F3/p63/p40-positive cells in WTs are aberrantly induced. Despite the abluminal location and p63/p40-positivity, these cells are unlikely to exhibit myoepithelial differentiation [37], considering they are negative for aSMA and calponin, representative myoepithelial markers.

Although studies on FOXI1 expression in tumors have not yet addressed the relationship with cellular damage, renal intercalated cells, and renal oncocytic neoplasms are often implicated. Renal intercalated cells are similar to ionocytes in that they are involved in ion exchange and are regulated by FOXI1. Renal oncocytic neoplasms have been found to express FOXI1, possibly associated with intercalated cells [25, 38–40]. These reports imply that FOXI1 expression and oncocytic features of neoplasms in different organs might be correlated. However, this hypothetical relationship might not always be true because oncocytic neoplasms other than WTs in our study did not express FOXI1. Instead, we speculate that FOXI1 is a marker limited to ionocytes and renal intercalated cells.

The observed mutually exclusive expression of two master regulators, FOXI1 and POU2F3, in different cell types in WTs seems unusual for a true (i.e., clonal) neoplasm and is consistent with the idea that WT is a reactive lesion. Indeed, the other salivary tumors, including those consisting of two (luminal and abluminal) cell types, did not exhibit this unique staining pattern. Interestingly, at least partly, the POU2F3-positive cells in WTs coexpressed BCL2 and KIT. To our knowledge, only one study pointed out BCL2 expression of WT cells with basal cell differentiation [41], and only one cytological study reported KIT expression in 75% of WTs [42]. Because KIT and BCL2 are often highly expressed in POU2F3-positive tuft cell-like carcinomas [17, 19, 22], the same feature in WTs could suggest that non-neoplastic POU2F3-positive cells might express BCL2 and KIT via non-mutational, possibly epigenetic mechanisms that, in turn, might be smoking-related [43, 44]. There were no POU2F3-positive cells coexpressing BCL2 and KIT in non-neoplastic salivary glands in our IHC with limited samples.

Future studies should investigate the expression profiles of WTs at a single-cell level to clearly understand the properties of FOXI1-positive and POU2F3-positive cells. Other tumors that contain these cells, such as PAs and adenoid cystic carcinomas, will also be investigated further. We can speculate that the histological diversity of PAs might have qualitatively different populations of ionocytes and tuft cells. The result that POU2F3-positive cells were observed in the luminal side of neoplastic ducts in PA, unlike their preferential distribution in the abluminal side in WTs, suggests that the precursors of POU2F3-positive cells in PAs maintain the capacity for an orthotopic tuft cell differentiation.

Because WTs and PAs are benign and consistently harbored FOX1- and POU2F3-positive cells, the frequencies of FOXI1 and POU2F3 expression were significantly higher in benign tumors than malignant ones, although our cases were not chronologically selected in this study. Thus, the presence of FOXI1-positive cells may suggest benign tumors in salivary glands and be of differential diagnostic value. This finding may be beneficial for diagnosing epithelial-rich PAs, which are sometimes difficult to diagnose based on small biopsies. Although POU2F3-positivity might also indicate benign tumors, considering the significantly higher frequency in benign tumors, the facts that even aggressive salivary gland tumors can harbor POU2F3-positive, possibly tuft cells, and that tumor-associated tuft cells can influence cancer aggressiveness through paracrine mechanisms [45], POU2F3-positivity may provide new translational perspectives on the role of tuft cells in malignant salivary and nonsalivary tumors, rather than diagnostic aid.

Our study has several limitations, such as the relatively small number of cases and the lack of comprehensive expression profile analysis. Thus, future studies should be conducted using more cases and subtypes and/or comprehensive expression profiling to confirm these results. Nonetheless, we believe the present results can potentially advance our understanding of salivary gland neoplasms, especially WT, and will lay the foundation for future research.

## Data Availability

All data generated or analysed during this study are included in this published article and its supplementary information files.
